# Diversity of anopheline species and their *Plasmodium* infection status in rural Bandarban, Bangladesh

**DOI:** 10.1186/1756-3305-5-150

**Published:** 2012-07-27

**Authors:** Mohammad Shafiul Alam, Sumit Chakma, Wasif A Khan, Gregory E Glass, Abu Naser Mohon, Rubayet Elahi, Laura C Norris, Milka Patracia Podder, Sabeena Ahmed, Rashidul Haque, David A Sack, David J Sullivan, Douglas E Norris

**Affiliations:** 1 International Centre for Diarrhoeal Disease Research Bangladesh (icddr,b), Dhaka, Bangladesh; 2 Johns Hopkins Malaria Research Institute, Johns Hopkins Bloomberg School of Public Health, Baltimore, MD, 21205, USA

## Abstract

**Background:**

Historically, the Chittagong Hill Tracts (CHT) of Bangladesh was considered hyperendemic for malaria. To better understand the contemporary malaria epidemiology and to develop new and innovative control strategies, comprehensive epidemiologic studies are ongoing in two endemic unions of Bandarban district of CHT. Within these studies entomological surveillance has been undertaken to study the role of the existing anopheline species involved in the malaria transmission cycle throughout the year.

**Methods:**

CDC miniature light traps were deployed to collect anopheline mosquitoes from the sleeping room of the selected houses each month in a single union (Kuhalong). Molecular identification was carried out for available *Anopheles* species complexes. Circumsporozoite proteins (CSP) for *Plasmodium falciparum*, *Plasmodium vivax*-210 (Pv-210) and *Plasmodium vivax*-247(Pv-247) were detected by Enzyme-linked immunosorbent assay (ELISA) from the female anopheline mosquitoes. To confirm CSP-ELISA results, polymerase chain reaction (PCR) was also performed.

**Results:**

A total of 2,837 anopheline mosquitoes, of which 2,576 were female, belonging to 20 species were collected from July 2009 -June 2010. *Anopheles jeyporiensis* was the most abundant species (18.9%), followed by *An. vagus* (16.8%) and *An. kochi* (14.4%). ELISA was performed on 2,467 female mosquitoes of 19 species. 15 (0.6%) female anophelines belonging to eight species were found to be positive for *Plasmodium* infection by CSP-ELISA. Of those, 11 (0.4%) mosquitoes were positive for *P. falciparum* and four (0.2%) for Pv-210. No mosquito was found positive for Pv-247. *An. maculatus* (2.1%, 2/97) had the highest infection rate followed by *An. umbrosus* (1.7%, 2/115) and *An. barbirostris* (1.1%, 2/186). Other infected species were *An. nigerrimus*, *An. nivipes, An. jeyporiensis*, *An. kochi*, and *An. vagus*. Out of 11 *P. falciparum* CSP positive samples, seven turned out to be positive by PCR. None of the samples positive for Pv-210 was positive by PCR. In terms of abundance and incrimination, the results suggest that *An. maculatus*, *An. jeyporiensis* and *An. nivipes* play important roles in malaria transmission in Kuhalong.

**Conclusion:**

The findings of this study suggest that even in the presence of an insecticide impregnated bed-net intervention, a number of *Anopheles* species still play a role in the transmission of malaria. Further investigations are required to reveal the detailed biology and insecticide resistance patterns of the vector mosquito species in endemic areas in Bangladesh in order to assist with the planning and implementation of improved malaria control strategies.

## Background

In Bangladesh, malaria is most endemic in the highland areas bordering India and Myanmar. Of the 13 malaria endemic districts in Bangladesh, the Chittagong Hill Tracts (CHT) are composed of three districts; Rangamati, Bandarban and Khagrachhari, which contribute 80% of the country’s total malaria case burden [1]. *Plasmodium falciparum* and *P. vivax* are the two main parasites in CHT [1,2] although some sporadic reports of *P. malariae* and *P. ovale* have been reported recently [3,4]. Malaria incidence is seasonal in Bangladesh where the warm and wet months of May-October define the peak season and the dry and cooler months of November-April define the off season [5].

To date, 35 anopheline species have been reported in Bangladesh [6]. Of those, four species, *An. baimaii* (*dirus* D), *An. minimusAn. sundaicus* and *An. philippinensis* were incriminated as malaria vectors during the Malaria Eradication Programme (MEP) of the 1960’s. In the early 1990’s three additional species (*An. aconitusAn. annularis* and *An. vagus*) were incriminated following three respective outbreaks in flood plain areas of the country [7,8]. However, due to loss of forest habitat, the density of two ‘primary vectors’ (*An. baimaii* and *An. minimus)* were dramatically reduced in several sporadic entomological investigations carried out by the Malaria and Parasitic Disease Control Unit (M&PDC) (personal communication with N. Chaudhury, Senior Entomologist, M&PDC).

An entomological investigation carried out in three malaria endemic border areas of the country during the peak of the 2009 transmission season found seven *Anopheles* species harboring *Plasmodium* based on ELISA. Except for *An. philippinensis* and *An. vagus* five other species (*An. karwariAn. maculatusAn. barbirostrisAn. nigerrimus*, and *An. subpictus*), not previously incriminated in Bangladesh, were found to be infected [8]. From that study it was concluded that in the absence of recognized ‘primary malaria vectors’, other *Anopheles* species might play a significant role in continued malaria transmission in Bangladesh.

In collaboration with the Johns Hopkins Malaria Research Institute (JHMRI), icddr,b initiated a study to understand the epidemiology of malaria transmission in two unions (Kuhalong and Rajbila) of Bandarban district in mid 2009. The method has been described elsewhere [9]. The distribution of the anopheline fauna and the species’ roles in the malaria transmission cycle in the study areas during both the wet and dry season remain the main focus of the current entomological investigations. The aim of this paper is to report the faunal diversity and *Plasmodium* infection status of anopheline species that have been collected in the first year (July 2009- June 2010) of the entomological surveillance in Kuhalong.

## Methods

### Study area

Kuhalong (22° 12′ 45" N, 92° 9' 35" E) is located adjacent to the Bandarban town. Kuhalong was divided into 12 clusters, which had roughly equal numbers of households as part of the epidemiologic study. A description of Kuhalong and its clusters have been provided earlier [9]. In brief, the union has a total area of 79 sq km with a population of more than 11,390. Kuhalong is a hilly and forested area with an average elevation of 80 meters ranging up to 152 meters. Some other important features of Kuhalong include the presence of intricate branches of rivers, several streams, marshy lands and plant monoculture (teak and rubber). The land for plant monoculture was created by destroying natural forest. 99.5% of the inhabitants of the area have bed net coverage (untreated/insecticide treated). But when asked, 10% of the respondents admitted that they had not used a bed net the previous night (unpublished data).

### Collection of *Anopheles* mosquitoes

CDC miniature light trap (model 512, John W. Hock Inc, USA) was deployed for indoor mosquito collection from the sleeping room. Traps were placed for 12 hours (6 pm to 6 am). During the wet season 100 houses were selected randomly, based on elevation and were trapped. This was reduced to 50 houses in the off (dry) season. Later, when trapping started in the second Union (Rajbila), the number was fixed to 60 houses at each union (5 houses in each of 12 clusters) due to the available resources and personnel. Each house was later trapped once in a month throughout the season in order to obtain longitudinal information.

### Mosquito sample preparation

Mosquitoes were collected from the light traps using a battery-powered aspirator and were subsequently killed by chloroform. Anopheline mosquitoes were sorted and preserved temporarily in plastic tubes. After identifying the species at the field office in Bandarban each mosquito was preserved in a separate plastic vial (1.5 ml) labelled and capped with silica gel and cotton.

### Circumsporozoite protein (CSP) ELISA

The ELISA methods were described previously elsewhere [10,11]. ELISA distinguished between circumsporozoite protein (CSP) of *P. falciparum* and two distinct polymorphs of *P. vivax*: Pv-210 and Pv-247. In each test, field caught male *Anopheles* spp. was used as the negative control. The positive controls and monoclonal antibody (MAB) for the ELISAs were obtained from the Centers for Disease Control and Prevention, Atlanta, USA. The optical density (OD) was measured at 410 nm in a Bio-Rad ELISA plate reader, 60 minutes after adding the substrate [8]. A cut-off value at least twice the mean OD of the negative controls was considered as positive. ELISA positive samples in a given day were repeated the next day to confirm the result.

### PCR

Remaining mosquito lysates were used for DNA extraction using the QiaAmp DNA Minikit (Qiagen, Valencia, CA), following the tissue extraction protocol. Nested PCR was performed to detect *P. falciparum* and *P. vivax* infection from extracted mosquito DNA using the primers described by Snounou *et al.*[12]. PCR conditions were modified to adjust for low concentration of DNA from mosquito samples. 2 μL of the amplicon from the first step was used as template in the second step.

To confirm taxonomic identification of some *Anopheles* species, a confirmatory PCR was made available, based on availability of the established protocols. Protocols used were Phuc *et al.*[13] for species belonging to *An. minimus* and Myzomia Series, two different protocols of Walton *et al*. [14,15] for Annularis and Maculatus groups, Huong *et al.*[16] for *An. dirus* sibling species, and Goswami *et al.*[17] for the *An. culicifacies* complex, respectively. Protocols were followed with some modifications. DNA amplifications were done on S1000® Thermal Cycler (Bio-Rad Laboratories, Inc., Hercules, CA) and visualized under UV illumination after electrophoresis on ethidium bromide-stained 1.5% agarose gels along with invitrogen® 100 base pair (bp) molecular mass marker (Life technologies, NY, USA). Representative samples (DNA from homogenates) were sent for sequencing to the JHMRI. The list of primers is given in Additional file 1.

### Sequencing of mosquito specimens

The variable internal transcribed spacer 2 region (ITS2) was amplified with primers from the flanking 5.8 S and 28 S genes; ITS2A (5′-TGT GAA CTG CAG GAC ACA T-3′) and ITS2B (5′-ACC CCC TGA ATT TAA GCA TA-3′) [18]. Because sequencing reactions with ITS2A frequently failed, some samples were amplified and sequenced using the novel primer ITS2B1 (5′-GTC CCT ACG TGC TGA GCT TC-3′). This primer binds further downstream in the 28 S gene; such that the 3′ portions of products immediately upstream of the ITS2B binding site, could be sequenced. Each 25 μL reaction contained 1X PCR buffer, 200 μM each dNTP, 30 pmol each primer, 2 units Taq polymerase, and 1.0 μL DNA template. Products were amplified in a thermocycler (MJ Research, Watertown, MA, USA) using the following conditions: 2 minute initial denaturation at 94°C, 40 cycles of 30 seconds at 94°C, 30 seconds at 50°C, and 40 seconds at 72°C, and a 10 minute final extension at 72°C. PCR product size ranged from 480 bp to 847 bp. 5 μL of each PCR product was subjected to electrophoresis on a 2% agarose gel, stained with ethidium bromide, and visualized under UV illumination. The remainder of each successful PCR reaction was purified using a Qiaquick PCR prep kit (Qiagen, Valencia, CA). Products were sequenced in both directions using dye terminator chemistry on a 3730xl DNA Analyzer (Applied Biosystems, Foster City, CA) at the Johns Hopkins University School of Medicine Sequencing and Synthesis Facility.

## Results

**Figure 1:**
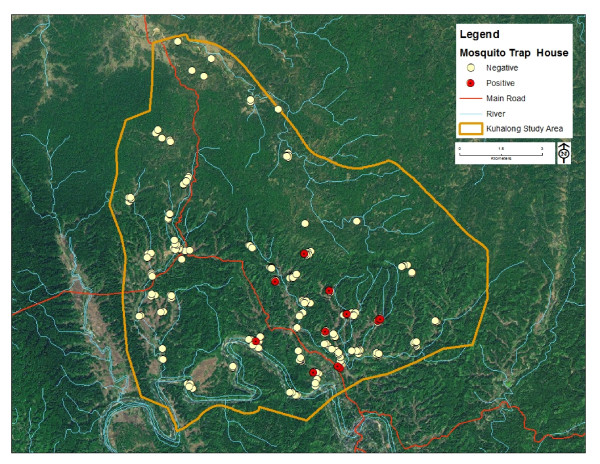
**A satellite image over study area showing location of 268 trapping houses.** Here, positive means houses that had CSP-positive mosquitoes and negative means houses with CSP-negative mosquitoes or without female anopheline mosquitoes.

Collections were made during 641 trap nights from July 2009 to June 2010 in households throughout Kuhalong (Figure 1). A total of 2,837 *Anopheles* mosquitoes (2,576 female and 261 male) were caught (4.4 mosquitoes/trap night; std. error 0.21). Among the collected female anophelines, 20 species were confirmed based on taxonomic characteristics, adjusted by PCR for available species complexes. A representative half of the specimens belonging to *An. philippinensis* based on morphological characteristics were later confirmed as *An. nivipes* by PCR followed by sequencing. Similarly the identity of all *An. pallidus* samples (approximately 30 specimens) was revised to *An. nivipes* after molecular confirmation. Therefore, all individual specimens originally morphologically identified as *An. philippinensis* and *An. pallidus* were listed as *An. nivipes* in our final data set. A number of *An. minimus* were molecularly identified as *An. varuna*, whereas the remaining *An. minimus* specimens were confirmed as *An. minimus* subspecies A. Three subspecies of *An. culicifacies* (B, C and E) were confirmed, with dominance of subspecies B. A strong concordance was observed between morphological and moleculer detection for *An. baimaii* and *An. maculatus* group.

**Figure 2:**
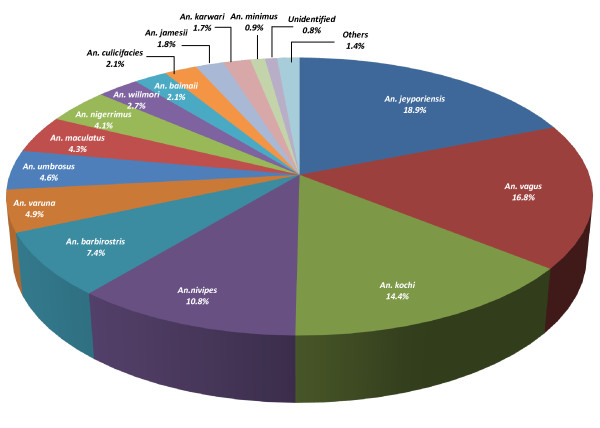
**Overall contribution of female*Anopheles*species in Kuhalong.**

**Figure 3:**
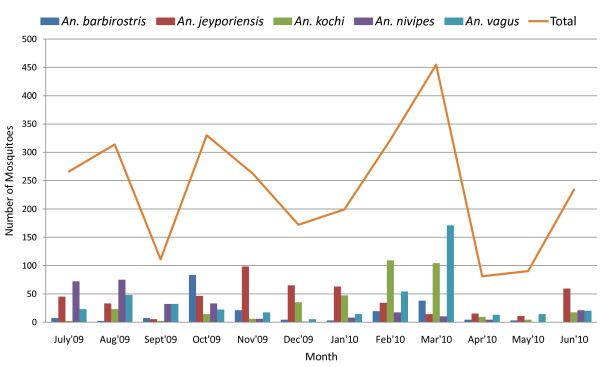
**Monthly frequency of five prevalent female*Anopheles*species in Kuhalong**

**Figure 4:**
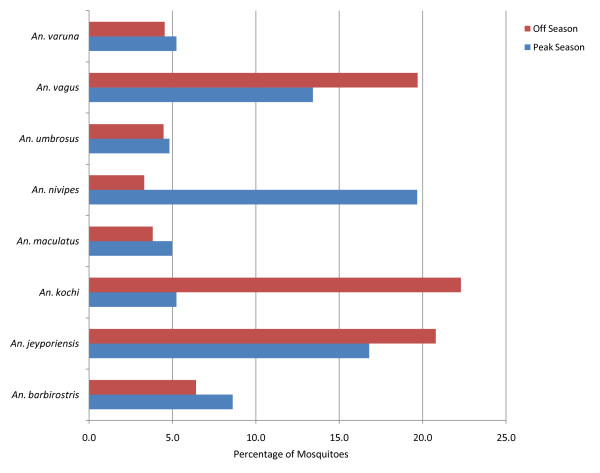
**Contribution of eight prevalent female*Anopheles*species according to transmission season**

Three species were nearly co-dominant. *An. jeyporiensis* (18.9%) was the most commonly captured species, followed by *An. vagus* (16.8%) and *An. kochi* (14.4%) (Figure 2). The relative abundance and dominance of anopheline species varied temporally throughout the year (Figure 3). Slightly more female anophelines were caught in the non-malaria dry season (n = 1391, mean 4.3 mosquitoes/trap night) than peak wet transmission season (n = 1185, mean 3.7 mosquitoes/trap night). However, this seasonal difference in mosquito collection rates was not statistically significant (p>0.05). The highest diversity of species was found in July 2009 (16) and lowest in September 2009 and April 2010 (11). *An. nivipes* appeared to be the dominant species during peak malaria season. However, in the off season, *An. kochi* was the dominant species. In both seasons, *An. jeyporiensis* and *An. vagus* were the second and third most numerous species, respectively. The density of *An. nivipes* complex was very low in the off season. *An. maculatus*, *An. nigerrimus*, *An. umbrosus* and *An. barbirostris* appeared to retain a moderate density throughout the year (Figure 4).

CSP-ELISA was performed on 2,467 female anopheline mosquitoes identified to 19 species. The untested mosquitoes (n = 109), including a single specimen of *An. turkhudi,* were kept as voucher specimens. Fifteen mosquitoes collected from 13 houses and belonging to eight species were *Plasmodium*-positive by CSP-ELISA. Thus, the overall infection rate was 0.6% (15/2467). By species, the highest infection rate was observed in *An. maculatus* (2.1%) followed by *An. umbrosus* (1.7%), *An. barbirostris* (1.1%), *An. nigerrimus* (1%), *An. nivipes* (0.8%), *An. jeyporiensis* (0.6%), *An. kochi* (0.5%), and *An. vagus* (0.2%), respectively (Table 1).

**Table 1:** Prevalence of female anopheline mosquitoes and their CSP positive rates

**Sl**	**Species**	**No. collected**	**No. tested**	**Positive**	**Pre (%)**
1	*An. aconitus*	19	18	0	0
2	*An. annularis*	2	2	0	0
3	*An. baimaii*	55	44	0	0
4	*An. barbirostris*	191	186	2	1.1
5	*An. culicifacis*	54	54	0	0
6	*An. jamesii*	47	45	0	0
7	*An. jeyporiensi*	488	479	3	0.6
8	*An. karwari*	45	42	0	0
9	*An. kochi*	372	369	2	0.5
10	*An. maculatus*	112	97	2	2.1
11	*An. minimus*	24	18	0	0
12	*An. nigerrimus*	105	104	1	1
13	*An. nivipes*	279	264	2	0.8
14	*An. subpictus*	8	6	0	0
15	*An. tessellatus*	7	6	0	0
16	*An. turkhudi*	1	0	0	0
17	*An. umbrosus*	119	115	2	1.7
18	*An. vagus*	433	429	1	0.2
19	*An. varuna*	125	121	0	0
20	*An. willmori*	70	68	0	0
21	Unidentified	20	0	0	0
	Total	2576	2467	15	0.6

Among the CSP-positive anophelines, 11 (0.4%) mosquitoes belonging to six species were found positive for *P. falciparum* and four (0.2%) mosquitoes belonging to three species were found positive for Pv-210 (Table 2). None of the mosquitoes were positive for Pv-247 or had mixed *Plasmodium* infection. *P. falciparum* positive *Anopheles* species included *An. barbirostris, An. jeyporiensis, An. kochi*, *An. maculatus, An. nigerrimus,* and *An. nivipes*. Pv-210 positive species included *An. nivipes, An. umbrosus* and *An. vagus*.

**Table 2:** **Summary information of CSP positive*Anopheles*female mosquitoes from Kuhalong, Bandarban**

**ID**	**Species Name**	**CSP ELISA**^a^	**PCR**^b^	**Fed**^c^
An-638	*An. nigerrimus*	Pf	Pf	0
An-677	*An. maculatus*	Pf	Pf	0
09-0064	*An. maculatus*	Pf	Pf	0
09-0376	*An. jeyporiensis*	Pf	Pf	1
09-0380	*An. nivipes*	Pf	Pf	0
09-0381	*An. kochi*	Pf	0	1
09-0396	*An. vagus*	Pv-210	0	0
09-0439	*An. barbirostris*	Pf	Pf	0
09-0459	*An. umbrosus*	Pv-210	0	1
09-0775	*An. jeyporiensis*	Pf	0	0
09-0785	*An. umbrosus*	Pv-210	0	1
09-0826	*An. barbirostris*	Pf	0	0
10-1147	*An. jeyporiensis*	Pf	Pf	0
10-1700	*An. kochi*	Pf	0	0
10-1917	*An. nivipes*	Pv-210	0	1

^a^*Pf* = *P. falciparum*, Pv-210 = *P. vivax*-210.

^b^*Pf* = *P. falciparum*(+ve), 0 = negative.

^c^*0* = unfed, 1 = blood fed.

**Figure 5:**
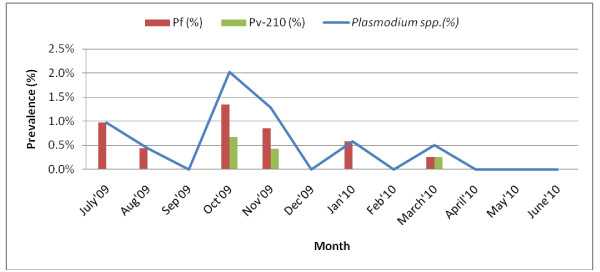
Monthly variation in CSP positive rates among anopheline species in Kuhalong

The highest monthly infection rates for *Plasmodium* were observed in October 2009 (2%) and November 2009 (1.3%). *Plasmodium-*infected mosquitoes were not detected in September or December of 2009 and April-June 2010 (Figure 5). In the dry season three sporozoite positive mosquitoes were caught in November 2009 and two in March 2010. Although the infection rate in peak season (0.8%) was twice that of off season (0.4%), this seasonal difference in mosquito infection rates was not statistically significant (p>0.05).

Of the 15 *Plasmodium*-positive mosquitoes, seven of these were positive for the presence of *P. falciparum* by nested PCR out of 11 that tested positive by CSP-ELISA. However, none of the Pv-210 CSP positive samples were positive by PCR. Species that had both CSP and PCR positive samples included five species: *An. maculatus*, *An. jeyporiensis*, *An. nivipes*, *An. barbirostris* and *An. nigerrimus* (Table 2).

## Discussion

The *Anopheles* species diversity is very high in Kuhalong. This diversity is even higher than what had been observed in a previous study in Matiranga, a sub-district of CHT, where 15 anopheline species were reported in a peak transmission season, of which seven were positive for CSP [8]. The greater number of anopheline species collected in Kuhalong might be due to the cumulative year-long trapping effort, as several species were most frequently sampled during the low transmission season (Figures 3 and 4). This highlights the importance of off season trapping.

Of the eight *Plasmodium*-infected anopheline species reported in this study, *An. jeyporiensis* and *An. kochi* have never been previously incriminated as vectors in Bangladesh. However, *An. jeyporiensis* and *An. kochi* have been considered to be potential vectors in the foothills of Assam, a north-eastern Indian state [19]. There is also a report of sporozoites in *An. jeyporiensis* made in 1944 by Macan from mosquitoes collected on the Myanmar-Bangladesh border [20]. The high relative abundance of *An. jeyporiensis* and its infection confirmed by CSP followed by PCR indicates that this species likely plays an important role in the malaria transmission in the area.

Although *An. maculatus* is considered an important vector in Peninsular Malaysia and Southern Thailand [21,22], it was thought to be a less important species in Bangladesh earlier. This species was first found to be CSP infected in Bangladesh in recent times on the basis of a single positive mosquito out of seven tested [8]. Our results re-emphasize its potential importance as a vector in this region.

Despite expectations based on prior observations, no *An. baimaii* or *An. minimus s.l.* were found CSP-positive during this study in Kuhalong. Further study is required, but these observations may indicate that these species are less abundant, are locally less competent or that this reflects a foraging effect/trapping bias. Similarly, no *An. culicifacies,* a major malaria vector in some parts of India and Sri Lanka [23,24], were found to be infected with *Plasmodium* in Kuhalong. It is worth noting that *An. culicifacies* is primarily represented by species B in Kuhalong, a species considered to be a non-vector or a poor vector [24,25], and it is common in the eastern states of India sharing borders with Bangladesh [26]. This supports the idea that this species does not have a vectorial role in Kuhalong. Apart from *An. vagus,* two other potential secondary malaria vectors in Bangladesh, *An. aconitus* and *An. annularis*[27,28], were rarely collected in Kuhalong and all were negative for *Plasmodium* infection by CSP ELISA.

*An. philippinensis* has historically been considered the main vector of malaria in the vast plain areas of Bangladesh, but has been considered rare in the CHT [7]. The findings of the present study, in accordance with other recent observations [8], initially suggested that this species has adapted to the high land areas and has expanded its role in the transmission of malaria. However, *An. nivipes* is morphologically very similar with *An. philippinensis* and often confused with the latter. The adults of these two species are differentiated by a single characteristic related to a pre-sector dark mark on the wing vein [29]. In the Assam State of India, recent studies document the role of the *An. philippinensis-nivipes* complex in malaria transmission [19,30] and later confirmed the presence of *P. falciparum* DNA in two *An. nivipes* specimens, that were molecularly identified [31]. The presence of *P. falciparum* specific CSP by ELISA and later parasite DNA in *An. nivipes* in the present study support the previous observations in India. These two species have never been differentiated in Bangladesh until this report.

Representatives of *An. barbirostris* and *An. nigerrimus* species were found to be positive for the presence of CSP, which was also seen in previous observations made in the CHT [8]. Previously in this region, *An. barbirostris* was confirmed as a vector in Sri Lanka based on CSP-ELISA [32].

Depletion of the forest habitats, preferred by *An. baimaii* and *An. minimus* and conversion to agricultural use may have allowed members of the *An. nivipes* to extend their range into highland areas. It is unknown how these species interact, whether it is the habitat that drives the relative abundance of these species or whether there is active competition and replacement as well*.* Thus, the bionomics of anopheline species requires further study and will reveal valuable information necessary for future control strategies.

As a method, CSP-ELISA has several advantages although it is thought to be less sensitive, particularly when low numbers of sporozoites are present [33]. Hence, a false positive result is also possible due to the presence of CSP in body parts other than salivary glands [33,34] and also due to the presence of bovine or swine blood [35]. On the other hand, PCR is more sensitive than ELISA and can detect fewer parasites [36], but is not sporozoite specific and is recommended to reconfirm CSP-ELISA results [37]. None of the Pv-210 CSP positive samples in the study tested positive by PCR. A low concentration of DNA obtained from CSP homogenates could be another reason for negative PCR results along with CSP false positive samples.

In terms of incrimination by overall abundance and infection rates, *An. maculatus, An. jeyporiensis* and *An. nivipes* all are likely to have important roles in malaria transmission in Kuhalong. This is not to say that other species do not also contribute significantly to malaria transmission in Kuhalong.

The National Malaria Control Programme (NMCP) of Bangladesh initiated a programme in 2007 with financial support from the Global Fund. This programme includes diagnosis and treatment, distribution of long-lasting insecticidal bed net (LLIN) and re-treatment of insecticide-treated nets (ITNs). As of 2010, the NMCP has treated/retreated more than 2 million bed nets with insecticides and distributed almost 1.7 million LLINs throughout the malaria endemic areas of the country [38]. However, no prominent work has been reported so far to monitor the resistance patterns of the anopheline mosquitoes against the insecticides used in these control efforts. The high diversity of anophelines, may increase the risk of insecticide resistance and could alter biting behaviours which may circumvent some of the protection from bednets. This high species diversity is typical of Southeast Asia, in contrast to many parts of Africa where only a few species are major contributors to *Plasmodium* transmission.

## Conclusions

The findings of this study illustrate that even in areas of Bangladesh where ITNs and LLINs have been deployed as malaria interventions, a number of diverse *Anopheles* species still play a role in the transmission of malaria. Further study of the bionomics, ecology, and insecticide resistance of these species is necessary to understand the transmission biology and to provide the information required for the development of evidence-based control programmes in Bangladesh. This insight may be useful to other countries similarly facing high burdens of malaria.

## Supplementary Material

Additional file 1 List of primers. Click here for file
