# Supplementary material for: Diversity of anopheline species and their Plasmodium infection status in rural Bandarban, Bangladesh
Source: Parasit Vectors. 2012 Jul 27;5:150. doi: 10.1186/1756-3305-5-150 (PMC3419674; doi:10.1186/1756-3305-5-150)
Supplement: Additional file 1 — List of primers. [file 1756-3305-5-150-S1.docx]

| **Sl** | **Anopheline Target group** | **Primer** | **Sequence (5'-3')** | **Product size (bp)** | **Reference** |
| --- | --- | --- | --- | --- | --- |
| 1 | Nested PCR for detecting Infection | rPLU 5 | CCTGTTGTTGCCTTAAACTTC |  | [16] |
| 2 |  | rPLU 6 | TTAAAATTGTTGCAGTTAAAACG |  |  |
| 3 |  | rFAL 1 | TTAAACTGG TTTGGGAAAACCAAATATATT | 212 |  |
| 4 |  | rFAL 2 | ACACAATGAACTCAATCATGACTACCCGTC |  |  |
| 5 |  | rVIV 1 | CGCTTCTAGCTTAATCCACATAACTGATAC | 120 |  |
| 6 |  | rVIV 2 | ACTTCCAAGCCGAAGCAAAGAAAGTCCTTA |  |  |
| 7 | *Anopheles* *minimus* and Myzomyia series | 5.8S | ATCACTCGGCTCATGGATCG |  | [17] |
| 8 |  | MIA | GGGCGCCATGTAGTTAGAGTTG | 184 |  |
| 9 |  | MIC | GGTTGCCCACTCAATACGGGTG | 509 |  |
| 10 |  | JEY | CTCCCCATAGCGCGTAAGC | 346 |  |
| 11 |  | VAR | GTGGCCCCGCAATGTATG | 252 |  |
| 12 |  | ACO | AGGTTCACCCCGCTCTGG | 306 |  |
| 13 | *Anopheles annularis* Group | 5.8F(ANN) | TGTGAACTGCAGGACACATG |  | [18] |
| 14 |  | PHI | GCACGCCATTAT GCGACAAAC | 166 |  |
| 15 |  | NIV | CATGTACCTCACGATACATGTA | 246 |  |
| 16 |  | ANN | ACCCCTTTGCTAGCGCCCGTG | 387 |  |
| 17 |  | PAL | GTTAAGTGAGACGATAAAGACC | 463 |  |
| 18 | *Anopheles maculatus* Group | 5.8F(MAC) | ATCACTCGGCTCGTGGATCG |  | [19] |
| 19 |  | MAC | GACGGTCAGTCTGGTAAAGT | 180 |  |
| 20 |  | PSEU | GCCCCCGGGTGTCAAACAG | 203 |  |
| 21 | *Anopheles dirus* sibling species | FD1 | AGGGCACAAAAGTTATTAACTT | 172 | [20] |
| 22 |  | RD1 | GTGAAGAGCGAATATTGTAGC |  |  |
| 23 | *Anopheles culicifacies* Complex | ADF | CTAATCGATATTTATTACAC | 359 | [21] |
| 24 |  | ADR | TTACTCCTAAAGAAGGC |  |  |
| 25 |  | DF | TTAGAGTTTGATTCTTAC | 166+359 |  |
| 26 |  | BCEF | AAATTATTTGAACAGTATTG |  |  |
| 27 |  | BCR | TTATTTATTGGTAAAACAAC | 248 |  |
| 28 |  | CR | AGGAGTATTAATTTCGTCT | 95+248 |  |
| 29 |  | ER | GTAAGAATCAAATTCTAAG | 178+248 |  |
